# The Genome Sequences of 90 Mushrooms

**DOI:** 10.1038/s41598-018-28303-2

**Published:** 2018-07-02

**Authors:** Huiying Li, Surui Wu, Xiao Ma, Wei Chen, Jing Zhang, Shengchang Duan, Yun Gao, Ling Kui, Wenli Huang, Peng Wu, Ruoyu Shi, Yifan Li, Yuanzhong Wang, Jieqing Li, Xiang Guo, Xiaoli Luo, Qiang Li, Chuan Xiong, Honggao Liu, Mingying Gui, Jun Sheng, Yang Dong

**Affiliations:** 1 Kunming University of Science and Technology, Kunming, 650500 Yunnan, China; 2 College of Biological Big Data, Yunnan Agriculture University, Kunming, 650201 Yunnan China; 3 Kunming Edible Fungi Institute of All China Federation of Supply and Marketing Cooperatives, Kunming, 650032 Yunnan China; 4 Yunnan Plateau Characteristic Agricultural Industry Research Institute, Kunming, 650201 Yunnan China; 5 Key Laboratory of Puer Tea Science, Ministry of Education, Yunnan Agricultural University, Kunming, 650201 Yunnan China; 6 Nowbio Biotechnology Company, Kunming, 650201 Yunnan China; 7 State Key Laboratory of Genetic Resources and Evolution, Kunming Institute of Zoology, Chinese Academy of Sciences, Kunming, 650223 Yunnan China; 8 Kunming College of Life Science, University of Chinese Academy of Sciences, Kunming, 650204 Yunnan China; 9 College of Agronomy and Biotechnology, Yunnan Agricultural University, Kunming, 650201 Yunnan China; 10 State Key Laboratory for Conservation and Utilization of Bio-Resources in Yunnan, Yunnan Agricultural University, Kunming, 650201 Yunnan China; 11 Key Laboratory for Agro-biodiversity and Pest Control of Ministry of Education, Yunnan Agricultural University, Kunming, 650201 Yunnan China; 12 Biotechnology and Nuclear Technology Research Institute, Sichuan Academy of Agricultural Sciences, Chengdu, 610061 Sichuan China

**Keywords:** Evolution, Genome

## Abstract

Macrofungus is defined as the fungus that grows an observable sporocarp. The sporocarps of many species are commonly called mushrooms and consumed by people all around the world as food and/or medicine. Most macrofungi belong to the divisions Basidiomycetes and Ascomycetes, which are estimated to contain more than 80,000 species in total. We report the draft genome assemblies of macrofungi (83 Basidiomycetes species and 7 Ascomycetes species) based on Illumina sequencing. The genome sizes of these species ranged from 27.4 Mb (*Hygrophorus russula*) to 202.2 MB (*Chroogomphus rutilus*). The numbers of protein-coding genes were predicted in the range of 9,511 (*Hygrophorus russula*) to 52,289 (*Craterellus lutescens*). This study provides the largest genomic dataset for macrofungi species. This resource will facilitate the artificial cultivation of edible mushrooms and the discovery of novel drug candidates.

## Introduction

The uncountable and diverse macrofungi species in the world are valuable resources for the discovery of novel drug candidates. For instance, a PTP1B inhibitor, (24E)-3,4-seco-cucurbita-4,24-diene-3-hydroxy-26,29-dioic acid, is extracted from the sporocarps of *Russula lepida*, and has potential uses in treating type-2 diabetes and obesity^1^. Metabolites of many *Lactarius* species have potential antitumor and antiviral activities^2^. *Auricularia auricula* polysaccharides were reported to have potent antioxidant activities against hydroxyl and superoxide radicals^3^. Despite the importance in drug discovery, the majority of macrofungi species could not be thoroughly researched in the laboratory partly due to the lack of reference genomes. So far, since a few macrofungi genomes have been reported^4–7^, many large fungal genome projects are in progress^8–10^. This reports the draft genome assemblies of 90 fungus, most of which are wild edible mushrooms (except *Annulohypoxylon stygium, Tricholoma bakamatsutake*, and *Russula foetens*). Our samples contained 83 Basidiomycetes species and 7 Ascomycetes species.

## Result

### Genome assembly and evaluation of the completeness of genome assembl**y**

We used platanus to assemble all genomes^11^. The sizes of assembled genomes ranged from 27.4 Mb (*Hygrophorus russula*) to 202.2 MB (*Chroogomphus rutilus*). The contig N50 numbers of these assemblies were in the range of 2,846 bp to 697,803 bp. The scaffold N50 numbers of these assemblies were in the range of 3,350 bp to 1,760,261 bp. All detailed assembly benchmarks were summarized in Supplementary Table S1 and Supplementary Table S2. We also evaluated the completeness of the final assemblies using BUSCO^12^. The result shows that the proportions of complete BUSCOS of the 90 species were in the range of 69.3% to 98.6%. 78 fungal genomes had a complete BUSCO proportion larger than 80%. All related BUSCO results were shown in Supplementary Table S1.

### Gene annotation

We used multiple methods to annotate the protein-coding genes for all 90 genomes, including *de novo* predictions and homology-based predictions. For the *de novo* predictions, we performed Augustus^13^ analysis on the repeat-masked genome with parameters trained from *Coprinopsis cinerea*, GenScan^14^, glimmerHMM^15^, SNAP^16^ analysis with parameters trained from *Arabidopsis thaliana* on the repeat-masked genome. For homology based predictions, we used the protein sets of eight fungal species for every macrofungus genome (Please see Supplementary Table S2 for details). All the reference protein sets were obtained from Ensembl fungi (http://fungi.ensembl.org/index.html).

The result shows that the numbers of the protein-coding genes were mostly in the range of 9,511 to 39,074. *Craterellus lutescens* had over 50,000 predicted protein-coding genes about 52,289. The average protein-coding gene lengths were in the range of 924 bp to 1,741 bp. Detailed information for each fungus was presented in Supplementary Table S2.

### Gene family clustering analysis

**Figure 1:**
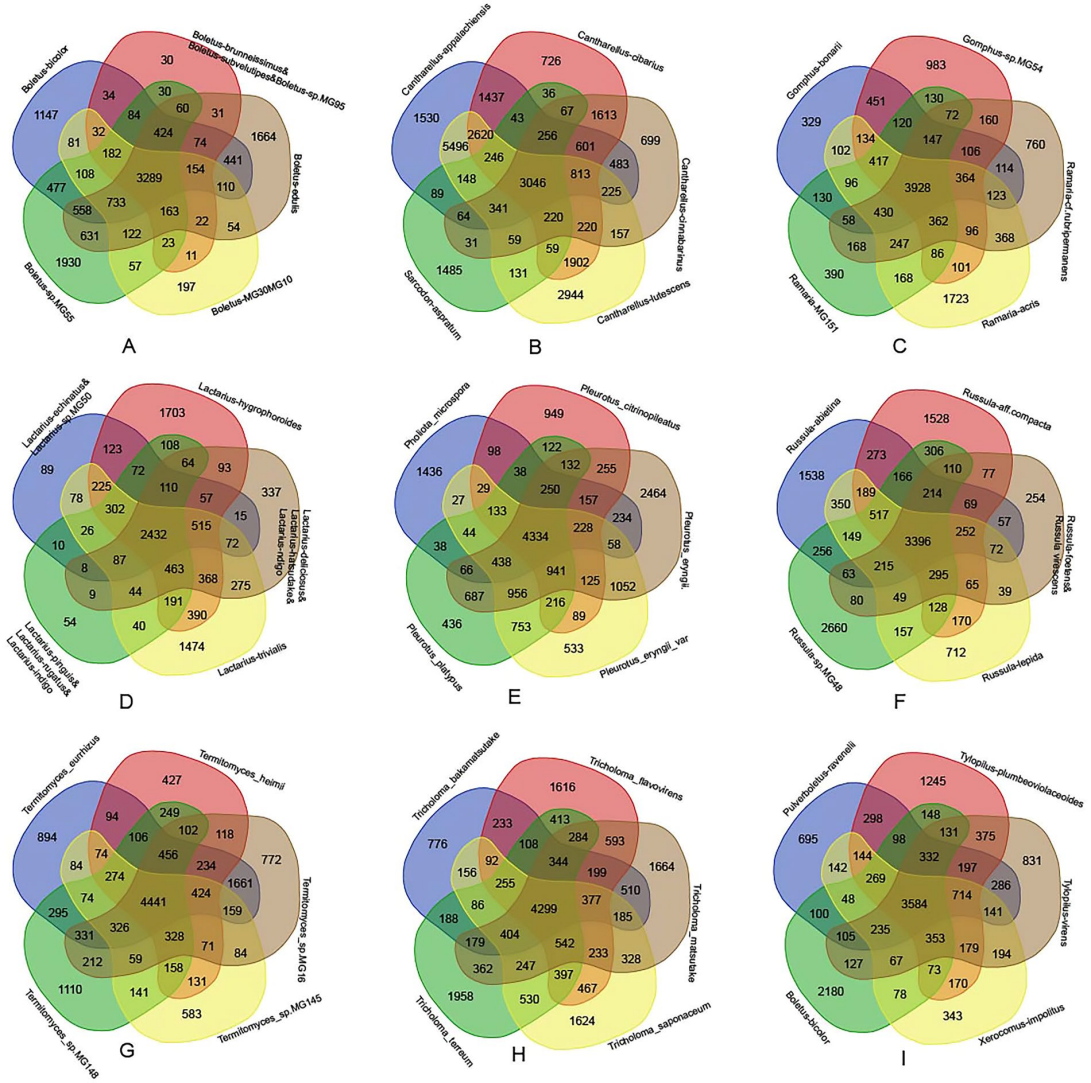
Venn diagram showing unique and shared gene families.

To identify and estimate the numbers of potential orthologous gene families, we applied the OrthoMCL (v. 2.0.9) pipeline^17^ using standard settings (BLASTP E-value < 1e^−5^) to compute the all-against-all similarities. The result was summarized in Venn diagram format using a web tool (http://bioinformatics.psb.ugent.be/webtools/Venn). All results were shown in Fig. 1. We arbitrarily grouped close-related mushroom species together for the analyses and found that each group had about 3,000~4,000 shared gene families.

### Phylogenetic Analysis

We then constructed phylogenetic trees for these macrofungi according to taxonomical divisions. For each phylogenetic tree, we used 8~10 reference fungi genomes. All single-copy orthologous genes identified in the gene family cluster analysis were used to construct a phylogenetic tree. MUSCLEv.3.8.31 with default settings was used to perform the multiple sequence alignments^18^. MrBayes^19^ was used to reconstruct phylogenetic trees. The result shown in Supplementary Fig. S1.

### Notes on CAZymes

Carbohydrate-active enzymes (CAZys) include carbohydrate esterases, glycoside hydrolases (GHs), glycosyltransferases (GTs), and polysaccharide lyases (PLs). We annotated the putative CAZy genes in all mushroom genomes by hmmer3.1^20^ against dbCAN-fam-HMMs.txt.v6 (http://csbl.bmb.uga.edu/dbCAN/^21^) and filtered the result with E < 1e-5. In general, *Craterellus lutescens* has a large number of CAZy genes compared with other species. *Morchella eximia* has a larger number of GH genes and *Cantharellus appalachiensis* has a larger number of GT genes than others except *Craterellus lutescens*.

### Analysis of Microsatellites

Microsatellites, also known as simple sequence repeats (SSRs), are composed of 1 to 6 nucleotide repeats in tandem. These genomic features contain important information of phenotypic diversity and genome organization^22^. We used MISA^23^ to identify mono- to hexa-nucleotide microsatellite motifs by default parameters. The results are shown in Supplementary Table S3. The numbers of SSRs range from 1,222 (*Laetiporus sulphureus*) to 30,904 (*Tuber calosporum*).

## Discussion

We believe this genome dataset will be a useful tool for various molecular investigations to promote biology-based medicine and agriculture research. It will also support the investigation of physiological characteristics, morphological differences, evolutionary, and metabolic analyses in comparative genomics, thereby providing evidence for population genetics of the many fungal species.

## Method

### Genomic DNA sequencing on Illumina platforms

All mushroom samples were obtained from the local fresh market in Yunnan and Sichuan provinces. To prevent contamination, we removed the surface with a sterile knife and took the middle part as the experimental material. We identified the mushrooms by observing the morphological characteristics and matching the ITS sequence against the database to determine the species. We provided the Mycobank accession numbers^24^ of all species in Supplementary Table S4, with which readers could get more information about the mushrooms in Mycobank.

About 400 mg sporocarp tissues from each sample were used to extract genomic DNA using the Plant Genomic DNA Extraction Kit DP320 (TIANGEN, Beijing, China). Paired-end libraries with insert sizes of 425 bp and 725 bp were constructed using the Next Ultra^TM^ DNA Library Prep Kit for Illumina (NEB, USA) according to manufacturer’s instructions, and subsequently sequenced on a HiSeq. 4000 platform (Illumina, USA) using the PE-150 module^25^. To improve the assembly quality, we filtered out the low-quality reads following these criteria: (1) Filter reads in which more than 5 percent of bases were N or poly A; (2) Filter low-quality reads in which more than 30 bases were low quality; (3) Filter reads with adapter contamination; (4) Filter reads with small size; (5) Filter PCR duplicates.

### Estimation of genome sizes

For each macrofungus, clean reads obtained from the Illumina platform were subjected to 17-mer frequency distribution analysis with Jellyfish^26^. Analysis parameters were set at -k 17, and the final result was plotted as a frequency graph. Two distinctive modes could be observed from some distribution curves, suggesting a high degree of heterozygosity. We then used the following formula to predict the genome size: genome size = *k*-mer_Number/Peak_Depth. The predicted genome sizes ranged from 36 Mb to 301.4 Mb. This suggests that the sequencing data represents about 40 to 150-fold coverage of the genome. The detailed information for all 90 species is listed in Supplementary Table S5.

### Genome assembly and Reapeat annotation

We used platanus to assemble all genomes with default parameters^11^. We compared the assembled genome and predicted genome in Supplementary Fig. S2 and evaluated the completeness of the final assemblies using BUSCO^15^ with the fungi gene set.

For the transposable element annotation, we used RepeatMasker and RepeatProteinMasker^27^ against Repbase (v.18.07) to identify known repeats in the genome. Tandem Repeat Finder^28^ was used to identified tandem repeats. In addition, we used RepeatModeler and LTR FINDER^29^ to identify *de novo* evolved repeats in the genome. The total length of repeated sequences of genome are in the rage of 1.34% to 94.7%. The detailed results were shown in Supplementary Table S2.

### Gene annotation

For homology-based predictions: First, we used TBLASTN with parameters of ‘*E*-value = 1e^−5^’ to cutoff the query sequences. Then concatenated the result which corresponded to reference proteins and filtered low-quality records by Solar^30^ software. Genomic sequence of each reference protein was extended upstream and downstream by 2,000 bp to represent a protein-coding region. Use GeneWise software^31^ to predict gene structure contained in each protein region. Homology-based and *de novo* were merged to a comprehensive and non-redundant gene set by EVidenceModeler^32^.

### Non-coding RNA annotation

We used tRNAscan-SE (version 1.31)^33^ software with default parameters for eukaryote to get tRNA annotation. We also used BLASTN with parameters of ‘E-value = 1e-5’ based on homology information of yeast rRNAs to get rRNA annotation. The miRNA and snRNA genes were predicted by INFERNAL software (http://infernal.janelia.org, version 1.1) against the Rfam database (Release 11.0)^34^. Detailed information was presented in Supplementary Table S2.

### Data availability

The genome sequence have been uploaded in NCBI with the project ID PRJNA454572, Supplementary Table S6 provide the project ID of raw data in NCBI.

### Material availability

Genomic DNA samples of all 90 species have been deposited in the collection of Yunnan Edible Mushroom Research Initiative of the Yunnan Agricultural University in China.

## Electronic supplementary material

Figure S1

Figure S2

Table S1

Table S2

Table S3

Table S4

Table S5

Table S6
