# Author Correction: The Genome Sequences of 90 Mushrooms

**DOI:** 10.1038/s41598-020-63941-5

**Published:** 2020-05-18

**Authors:** Huiying Li, Surui Wu, Xiao Ma, Wei Chen, Jing Zhang, Shengchang Duan, Yun Gao, Ling Kui, Wenli Huang, Peng Wu, Ruoyu Shi, Yifan Li, Yuanzhong Wang, Jieqing Li, Xiang Guo, Xiaoli Luo, Qiang Li, Chuan Xiong, Honggao Liu, Mingying Gui, Jun Sheng, Yang Dong

**Affiliations:** 1 Kunming University of Science and Technology, Kunming, 650500 Yunnan China; 2 College of Biological Big Data, Yunnan Agriculture University, Kunming, 650201 Yunnan China; 3 Kunming Edible Fungi Institute of All China Federation of Supply and Marketing Cooperatives, Kunming, 650032 Yunnan China; 4 Yunnan Plateau Characteristic Agricultural Industry Research Institute, Kunming, 650201 Yunnan China; 5 Key Laboratory of Puer Tea Science, Ministry of Education, Yunnan Agricultural University, Kunming, 650201 Yunnan China; 6 Nowbio Biotechnology Company, Kunming, 650201 Yunnan China; 7 State Key Laboratory of Genetic Resources and Evolution, Kunming Institute of Zoology, Chinese Academy of Sciences, Kunming, 650223 Yunnan China; 8 Kunming College of Life Science, University of Chinese Academy of Sciences, Kunming, 650204 Yunnan China; 9 College of Agronomy and Biotechnology, Yunnan Agricultural University, Kunming, 650201 Yunnan China; 10 State Key Laboratory for Conservation and Utilization of Bio-Resources in Yunnan, Yunnan Agricultural University, Kunming, 650201 Yunnan China; 11 Key Laboratory for Agro-biodiversity and Pest Control of Ministry of Education, Yunnan Agricultural University, Kunming, 650201 Yunnan China; 12 Biotechnology and Nuclear Technology Research Institute, Sichuan Academy of Agricultural Sciences, Chengdu, 610061 Sichuan China

Correction to: *Scientific Reports* 10.1038/s41598-018-28303-2, published online 02 July 2018

In the original version of this Article, Huiying Li, Surui Wu and Xiao Ma were omitted as equally contributing authors. This has now been corrected in the PDF and HTML versions of the paper, and in the accompanying Supplemental Material.

