# Supplementary material for: The Genome Sequences of 90 Mushrooms
Source: Sci Rep. 2018 Jul 2;8:9982. doi: 10.1038/s41598-018-28303-2 (PMC6028375; doi:10.1038/s41598-018-28303-2)
Supplement: Supplementary file 1 — Figure S1 [file 41598_2018_28303_MOESM1_ESM.pdf]

The Genome Sequences of 90 Mushrooms

Huiying Li[1], Surui Wu[3,#], Xiao Ma[2,4,5,#], Wei Chen[2,4], Jing Zhang[6], Shengchang Duan[6], Yun Gao[6], Ling Kui[7,8], Wenli Huang[12], Peng Wu[2,4], Ruoyu Shi[2,4], Yifan Li [2,5], Yuanzhong Wang[9] , Jieqing Li[9] , Xiang Guo[3] , Xiaoli Luo[3], Qiang Li[12], Chuan Xiong[12], Honggao Liu[9], Mingying Gui[3*], Jun Sheng[2,4,*], Yang Dong[2,10,11,*]

[1]Kunming University of Science and Technology, Kunming, 650500, Yunnan, China.

[2]College of Biological Big Data, Yunnan Agriculture University,Kunming, 650201, Yunnan, China.

[3]Kunming Edible Fungi Institute of All China Federation of Supply and Marketing Cooperatives, Kunming , 650032, Yunnan, China

[4]Yunnan Research Institute for Local Plateau Agriculture and Industry, Kunming, 650201, Yunnan, China.

[5]Key Laboratory of Puer Tea Science, Ministry of Education, Yunnan Agricultural University, Kunming, 650201, Yunnan, China.

[6]Nowbio Biotechnology Company, Kunming, 650201, Yunnan, China.

[7]State Key Laboratory of Genetic Resources and Evolution, Kunming Institute of Zoology, Chinese Academy of Sciences, Kunming, 650223, Yunnan, China.

[8]Kunming College of Life Science, University of Chinese Academy of Sciences, Kunming 650204, Yunnan, China.

[9]College of Agronomy and Biotechnology, Yunnan Agricultural University, Kunming, 650201, Yunnan, China

[10]State Key Laboratory for Conservation and Utilization of Bio-Resources in Yunnan, Yunnan Agricultural University, Kunming, 650201, Yunnan, China.

[11]Key Laboratory for Agro-biodiversity and Pest Control of Ministry of Education, Yunnan Agricultural University, Kunming, 650201, Yunnan, China.

[12]Biotechnology and Nuclear Technology Research Institute, Sichuan Academy of Agricultural Sciences, Chengdu, 610061, Sichuan, China.

#Huiying Li, Surui Wu and Xiao Ma contributed equally.

Supplementary Figure S1:The phylogenetic trees of fungus.

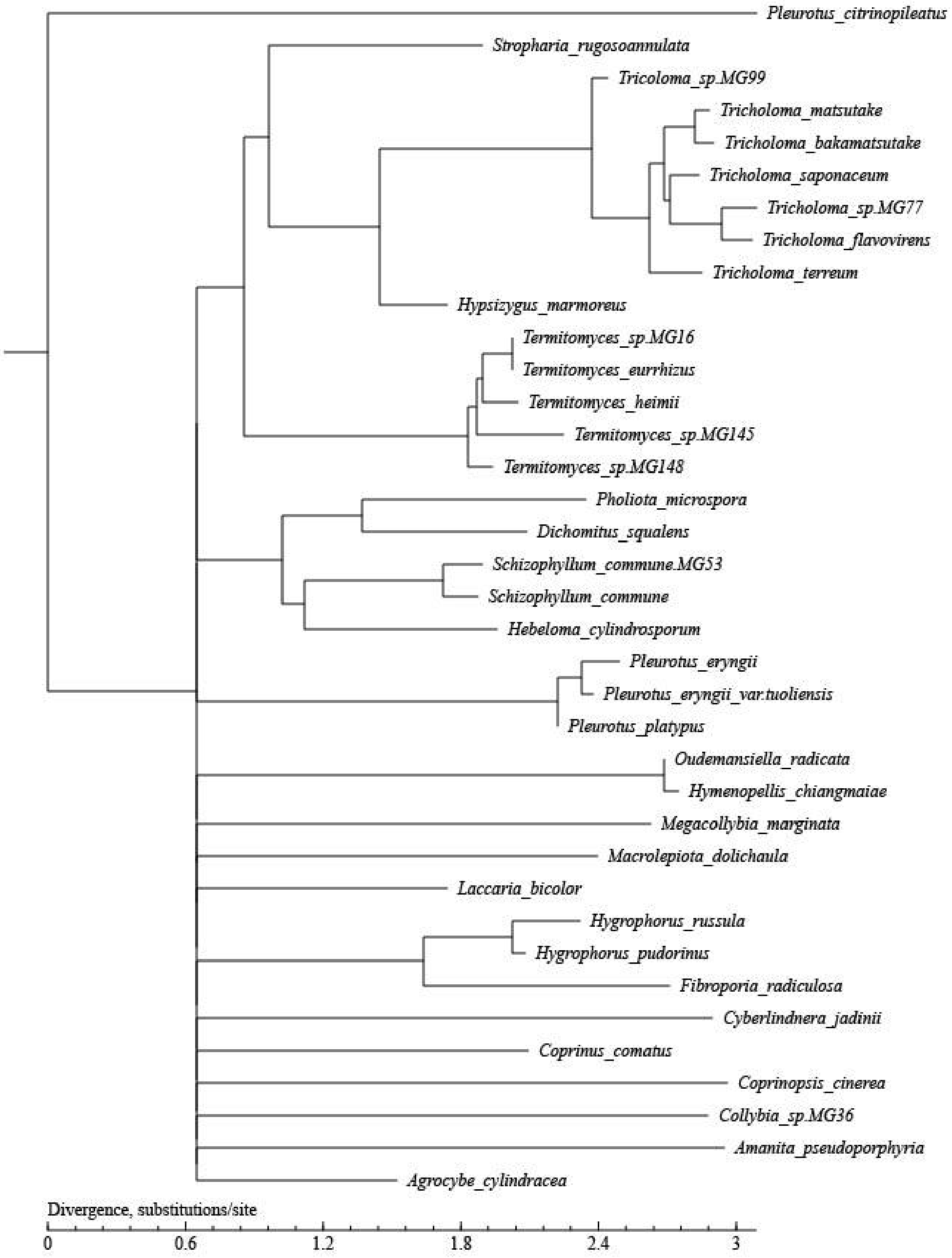

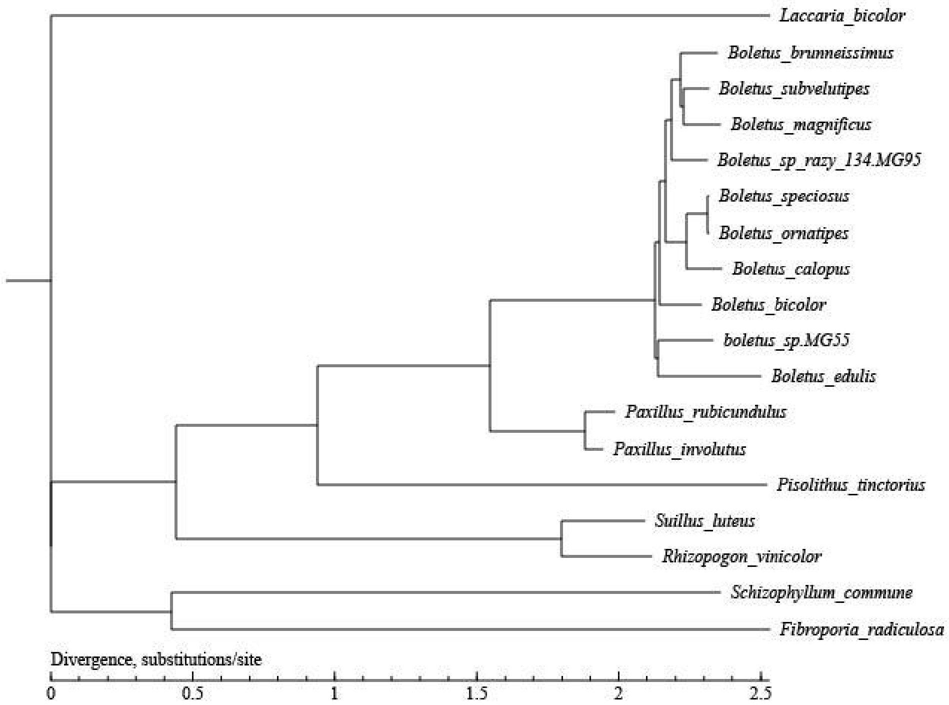
Laccaria_bicolor
Boletus_brunneissimus
Boletus_subvelutipes
Boletus_magnificus
Boletus_sp_razy_134.MG95
Boletus_speciosus
Boletus_ornatipes
Boletus_calopus
Boletus_bicolor
boletus_sp.MG55
Boletus_edulis
Paxillus_rubicundulus
Paxillus_involutus
Pisolithus_tinctorius
Suillus_luteus
Rhizopogon_vinicolor
Schizophyllum_commune
Fibroporia_radiculosa
Divergence, substitutions/site
0
0.5
1
1.5
2
2.5

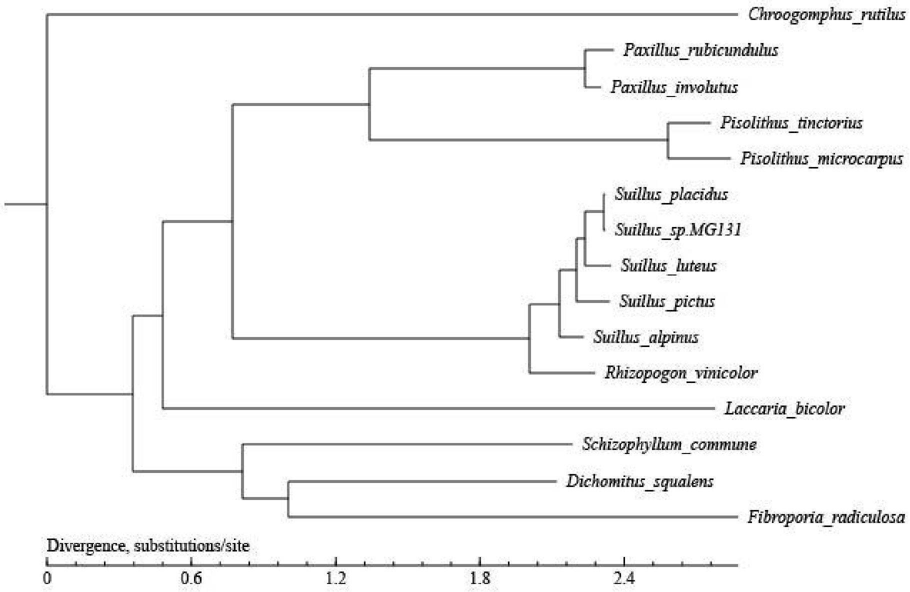
Chroogomphus_rutilus
Paxillus_rubicundulus
Paxillus_involutus
Pisolithus_tinctorius
Pisolithus_microcarpus
Suillus_placidus
Suillus_sp.MG131
Suillus_luteus
Suillus_pictus
Suillus_alpinus
Rhizopogon_vinicolor
Laccaria_bicolor
Schizophyllum_commune
Dichomitus_squalens
Fibroporia_radiculosa
Divergence, substitutions/site
0
0.6
1.2
1.8
2.4

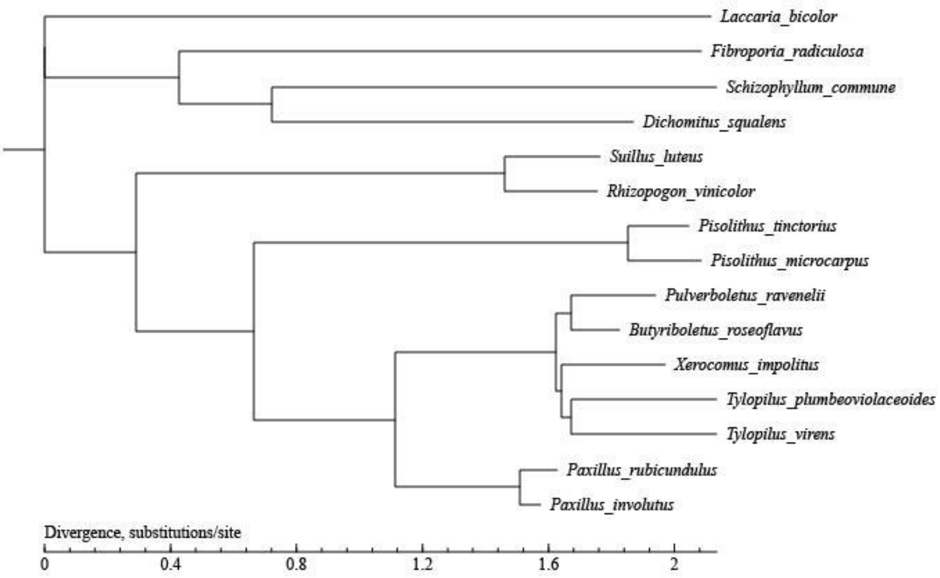
Laccaria_bicolor
Fibroporia_radiculosa
Schizophyllum_commune
Dichomitus_squalens
Suillus_luteus
Rhizopogon_vinicolor
Pisolithus_tinctorius
Pisolithus_microcarpus
Pulverboletus_ravenelii
Butyriboletus_roseoflavus
Xerocomus_impolitus
Tylopilus_plumbeoviolaceoides
Tylopilus_virens
Paxillus_rubicundulus
Paxillus_involutus
Divergence, substitutions/site
0
0.4
0.8
1.2
1.6
2

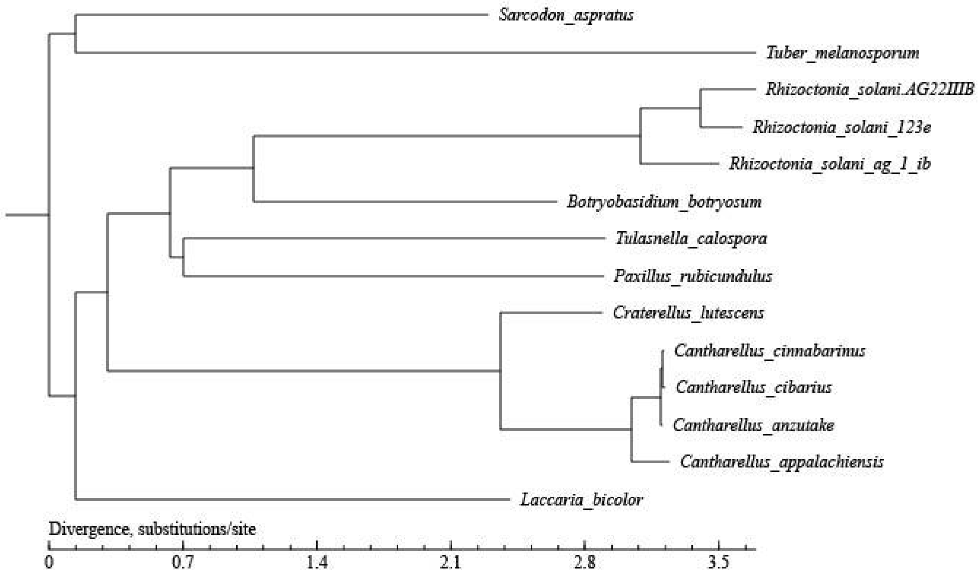
Sarcodon_aspratus
Tuber_melanosporum
Rhizoctonia_solani.AG22IIIB
Rhizoctonia_solani_123e
Rhizoctonia_solani_ag_1_ib
Botryobasidium_botryosum
Tulasnella_calospora
Paxillus_rubicundulus
Craterellus_lutescens
Cantharellus_cinnabarinus
Cantharellus_cibarius
Cantharellus_anzutake
Cantharellus_appalachiensis
Laccaria_bicolor
Divergence, substitutions/site
0
0.7
1.4
2.1
2.8
3.5

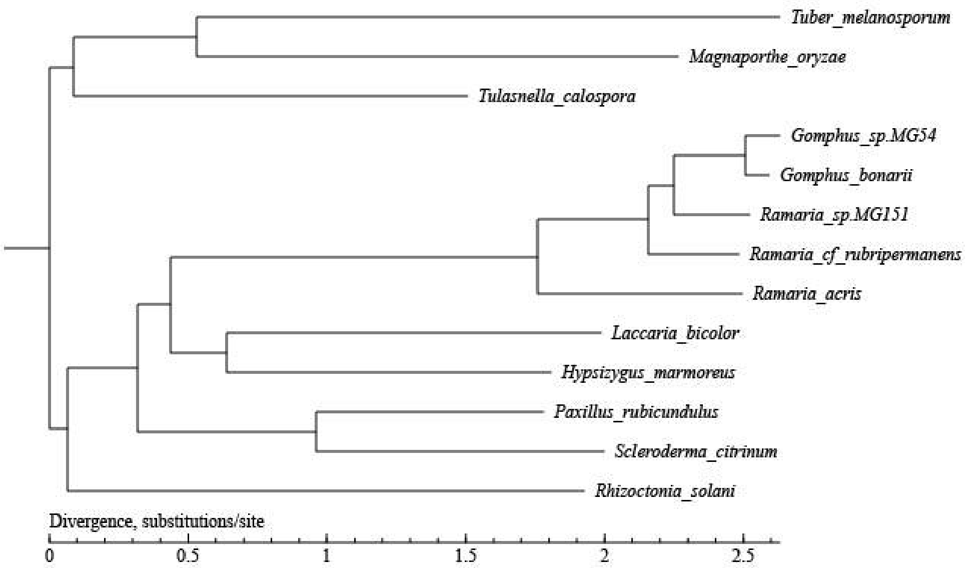
Tuber_melanosporum
Magnaporthe_oryzae
Tulasnella_calospora
Gomphus_sp.MG54
Gomphus_bonarii
Ramaria_sp.MG151
Ramaria_cf_rubripermanens
Ramaria_acris
Laccaria_bicolor
Hypsizygus_marmoreus
Paxillus_rubicundulus
Scleroderma_citrinum
Rhizoctonia_solani
Divergence, substitutions/site
0
0.5
1
1.5
2
2.5

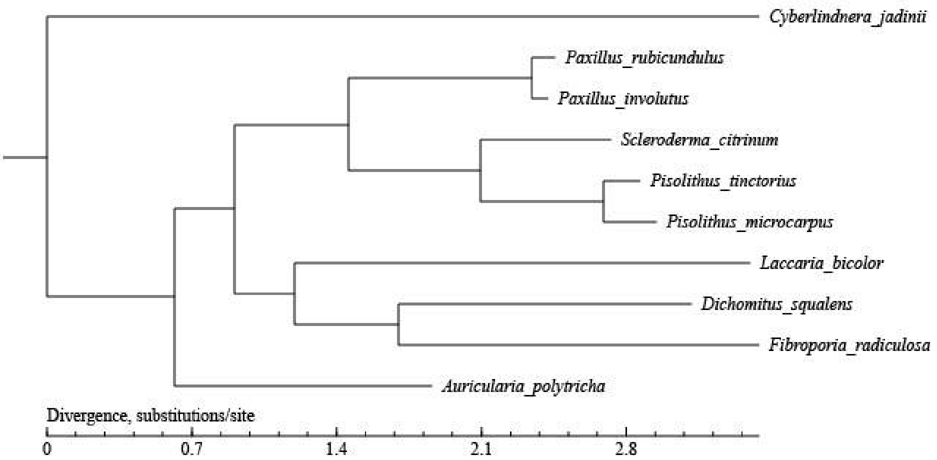
Cyberlindnera_jadinii
Paxillus_rubicundulus
Paxillus_involutus
Scleroderma_citrinum
Pisolithus_tinctorius
Pisolithus_microcarpus
Laccaria_bicolor
Dichomitus_squalens
Fibroporia_radiculosa
Auricularia_polytricha
Divergence, substitutions/site
0
0.7
1.4
2.1
2.8

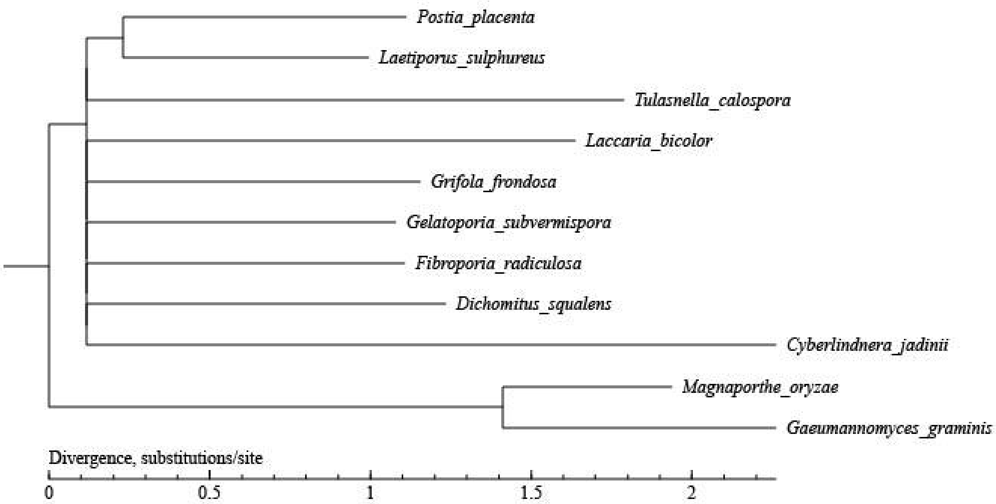
Postia_placenta
Laetiporus_sulphureus
Tulasnella_calospora
Laccaria_bicolor
Grifola_frondosa
Gelatoporia_subvermispora
Fibroporia_radiculosa
Dichomitus_squalens
Cyberlindnera_jadinii
Magnaporthe_oryzae
Gaeumannomyces_graminis
Divergence, substitutions/site
0
0.5
1
1.5
2

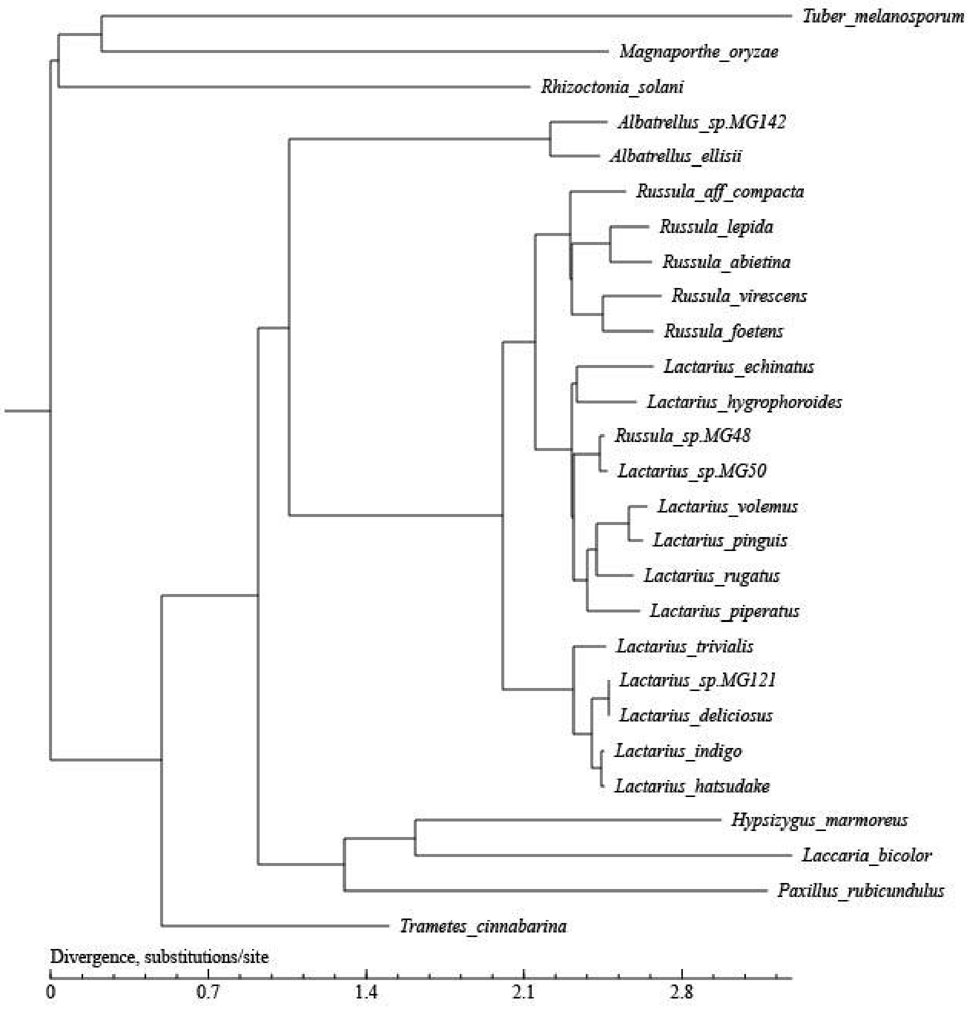
Tuber_melanosporum
Magnaporthe_oryzae
Rhizoctonia_solani
Albatrellus_sp.MG142
Albatrellus_ellisii
Russula_aff_compacta
Russula_lepida
Russula_abietina
Russula_virescens
Russula_foetens
Lactarius_echinatus
Lactarius_hygrophoroides
Russula_sp.MG48
Lactarius_sp.MG50
Lactarius_volemus
Lactarius_pinguis
Lactarius_rugatus
Lactarius_piperatus
Lactarius_trivialis
Lactarius_sp.MG121
Lactarius_deliciosus
Lactarius_indigo
Lactarius_hatsudake
Hypsizygus_marmoreus
Laccaria_bicolor
Paxillus_rubicundulus
Trametes_cinnabarina
Divergence, substitutions/site
0
0.7
1.4
2.1
2.8

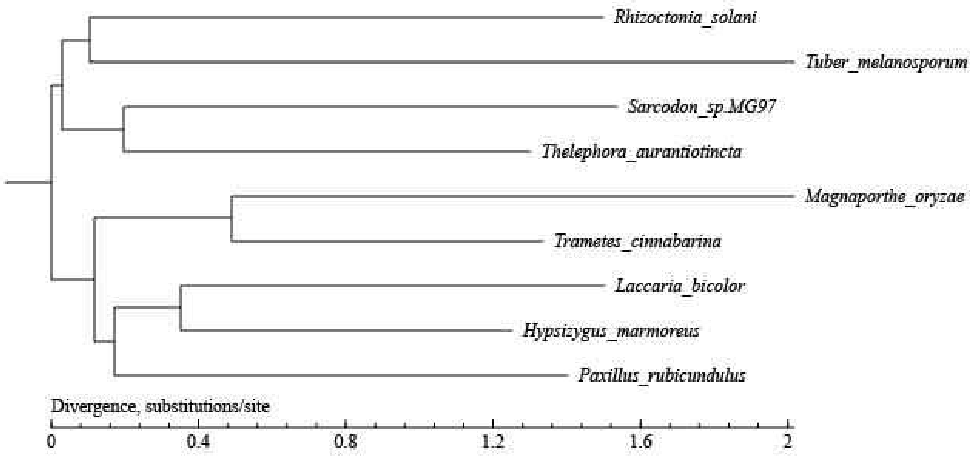
Rhizoctonia_solani
Tuber_melanosporum
Sarcodon_sp.MG97
Thelephora_aurantiotincta
Magnaporthe_oryzae
Trametes_cinnabarina
Laccaria_bicolor
Hypsizygus_marmoreus
Paxillus_rubicundulus
Divergence, substitutions/site
0
0.4
0.8
1.2
1.6
2

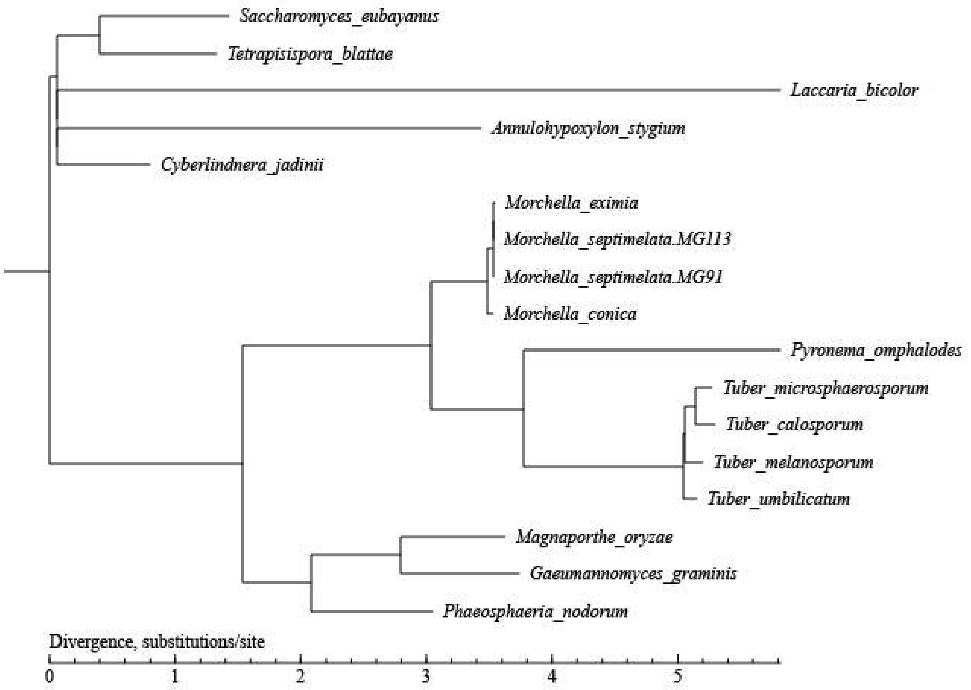
Saccharomyces_eubayanus
Tetrapisispora_blattae
Laccaria_bicolor
Annulohypoxylon_stygium
Cyberlindnera_jadinii
Morchella_eximia
Morchella_septimelata.MG113
Morchella_septimelata.MG91
Morchella_conica
Pyronema_omphalodes
Tuber_microsphaerosporum
Tuber_calosporum
Tuber_melanosporum
Tuber_umbilicatum
Magnaporthe_oryzae
Gaeumannomyces_graminis
Phaeosphaeria_nodorum
Divergence, substitutions/site
0
1
2
3
4
5
