# Supplementary material for: The Genome Sequences of 90 Mushrooms
Source: Sci Rep. 2018 Jul 2;8:9982. doi: 10.1038/s41598-018-28303-2 (PMC6028375; doi:10.1038/s41598-018-28303-2)
Supplement: Supplementary file 3 — Table S1 [file 41598_2018_28303_MOESM3_ESM.pdf]

The Genome Sequences of 90 Mushrooms

Huiying Li[1], Surui Wu[3,#], Xiao Ma[2,4,5,#], Wei Chen[2,4], Jing Zhang[6], Shengchang Duan[6], Yun Gao[6], Ling Kui[7,8], Wenli Huang[12], Peng Wu[2,4], Ruoyu Shi[2,4], Yifan Li [2,5], Yuanzhong Wang[9] , Jieqing Li[9] , Xiang Guo[3] , Xiaoli Luo[3], Qiang Li[12], Chuan Xiong[12], Honggao Liu[9], Mingying Gui[3*], Jun Sheng[2,4,*], Yang Dong[2,10,11,*]

[1]Kunming University of Science and Technology, Kunming, 650500, Yunnan, China.

[2]College of Biological Big Data, Yunnan Agriculture University,Kunming, 650201, Yunnan, China.

[3]Kunming Edible Fungi Institute of All China Federation of Supply and Marketing Cooperatives, Kunming , 650032, Yunnan, China

[4]Yunnan Research Institute for Local Plateau Agriculture and Industry, Kunming, 650201, Yunnan, China.

[5]Key Laboratory of Puer Tea Science, Ministry of Education, Yunnan Agricultural University, Kunming, 650201, Yunnan, China.

[6]Nowbio Biotechnology Company, Kunming, 650201, Yunnan, China.

[7]State Key Laboratory of Genetic Resources and Evolution, Kunming Institute of Zoology, Chinese Academy of Sciences, Kunming, 650223, Yunnan, China.

[8]Kunming College of Life Science, University of Chinese Academy of Sciences, Kunming 650204, Yunnan, China.

[9]College of Agronomy and Biotechnology, Yunnan Agricultural University, Kunming, 650201, Yunnan, China

[10]State Key Laboratory for Conservation and Utilization of Bio-Resources in Yunnan, Yunnan Agricultural University, Kunming, 650201, Yunnan, China.

[11]Key Laboratory for Agro-biodiversity and Pest Control of Ministry of Education, Yunnan Agricultural University, Kunming, 650201, Yunnan, China.

[12]Biotechnology and Nuclear Technology Research Institute, Sichuan Academy of Agricultural Sciences, Chengdu, 610061, Sichuan, China.

#Huiying Li, Surui Wu, Xiao Ma contributed equally.

Supplementary Table S1:Summary of genome assembly, gene number and statistics of the completeness of genome by BUSCO.

SPECIES	scaffoldN50(bp)	scaffold total (Mb)	gene number	busco result
Agrocybe cylindracea(MG21)	22390	54.9	22535	91.40%
Albatrellus ellisii(MG60)	11933	140.4	30685	88.90%
Albatrellus sp*(MG142)*	3350	63.1	11813	84.10%
Amanita pseudoporphyria(MG37)	6892	51.7	16864	73.50%
Annulohypoxylon stygium(MG137)	1760261	37	13781	98.60%
Auricularia polytricha(MG66)	37808	37.6	14118	88.20%
Boletus bicolor(MG1)	8949	46.6	14379	89.30%
Boletus brunneissimus(MG7)	9707	49.2	15497	84.80%
Boletus calopus(MG23)	43039	31.5	11429	89.70%
Boletus edulis(MG6)	11979	51.4	19907	82.70%
Boletus magnificus(MG22)	20939	38.9	13710	91.70%
Boletus ornatipes(MG30)	20819	28.6	12045	85.90%
Boletus sp*(MG55)*	8882	38.3	13261	86.20%
Boletus sp*(razy-134)(MG95)*	9493	53.7	20440	86.50%
Boletus speciosus(MG10)	30345	28.9	11861	89.70%
Boletus subvelutipes(MG31)	7746	50.1	15009	75.10%
Butyriboletus roseoflavus(MG29)	46028	27.7	11278	90.40%
Cantharellus appalachiensis(MG38)	11713	128.2	39074	81.80%
Cantharellus cibarius(MG75)	10540	62	24466	72.70%
Cantharellus cinnabarinus(MG28)	7906	61.6	15448	77.50%
Chroogomphus rutilus(MG62)	7873	202.2	18574	71.70%

Collybia sp*(MG36)*	9829	45.4	17905	84.10%
Coprinus comatus(MG80)	56281	29.7	10160	87.60%
Craterellus lutescens(MG144)	7694	160.3	52289	80.30%
Gomphus bonarii(MG147)	4149	125.7	12565	83.70%
Gomphus sp*(MG54)*	7345	201.5	18806	69.30%
Grifola frondosa (MG88)	16307	40.7	17458	84.20%
Hygrophorus pudorinus (MG65)	9252	77.1	21692	86.90%
Hygrophorus russula(MG78)	26194	27.4	9511	85.50%
Hymenopellis chiangmaiae(MG56)	17244	77.4	34771	94.50%
Lactarius deliciosus(MG9)	19116	54.2	12997	90.60%
Lactarius echinatus (razy-131)(MG122)	11743	47.1	13477	87.90%
Lactarius hatsudake(MG20)	5002	73.2	18513	84.50%
Lactarius hygrophoroides(MG19)	11062	54.5	14415	86.90%
Lactarius indigo(rll-109)(MG109)	12805	76.3	21539	88.60%
Lactarius sp*(rll-107)(MG121)*	12107	63.7	23531	89.30%
Lactarius pinguis(MG27)	18272	47.4	15740	84.20%
Lactarius piperatus(MG49)	15739	49.1	16161	82.10%
Lactarius rugatus(rmsh-101)(MG108)	5436	38.6	12848	79.70%
Lactarius sp*(MG50)*	12004	64.9	18611	85.50%
Lactarius trivialis (MG71)	33750	35.3	13584	91.00%
Lactarius volemus(MG8)	26839	42.4	13942	91.10%
Laetiporus sulphureus(MG138)	14705	50	22479	85.90%
Macrolepiota dolichaula(MG24)	15588	53	15034	85.10%
Megacollybia marginata(MG68)	14958	97	28041	87.90%

Morchella eximia (MG90)	45822	75	28143	83.40%
Morchella septimelata(MG113)	49531	47.6	12694	88.70%
Morchella septimelata(MG91)	27983	50.1	12778	91.40%
Oudemansiella radicata(MG139)	5949	68.9	30095	75.80%
Pholiota microspora (MG134)	21556	34	12595	88.90%
Pleurotus citrinopileatus(MG63)	9438	35.8	15237	82.10%
Pleurotus eryngii var.tuoliensis(MG79)	3856	174.6	16964	91.40%
Pleurotus eryngii(MG61)	21774	42.3	34918	93.50%
Pleurotus platypus(MG11)	59741	39	17105	92.80%
Pulveroboletus ravenelii(MG41)	12400	43.9	15403	81.40%
Ramaria cf. rubripermanens(MG17)	9879	84.5	16314	88.20%
Ramaria sp*(MG151)*	13453	82.8	12910	85.80%
Russula abietina(MG43)	10186	51.9	18455	80.70%
Russula aff. compacta(MG44)	7590	74.5	18437	87.50%
Russula foetens(MG47)	10045	48.4	16606	79.30%
Russula lepida(MG46)	45215	40.7	12860	93.10%
Russula sp*(MG48)*	12162	63.2	17178	85.50%
Russula virescens(MG14)	30016	61.8	18638	92.80%
Sarcodon aspratus(MG57)	30377	34.7	12541	90.00%
Sarcodon sp*(razy-129)(MG97)*	66766	31.1	9893	87.90%
Schizophyllum commune(MG53)	54684	49.1	27102	92.40%
Stropharia rugosoannulata (MG69)	8322	49.5	17683	87.60%
Suillus alpinus(MG64)	24119	37.3	14365	86.90%
Suillus pictus(MG42)	12430	88.5	22230	92.80%

Suillus placidus(MG34)	9146	39.7	15771	84.80%
Suillus sp*(MG131)*	14445	39.7	15863	84.50%
Termitomyces eurrhizus(MG13)	7395	84.5	18560	71.40%
Termitomyces heimii(MG15)	28644	54.8	11630	91.40%
Termitomyces sp*(MG145)*	62924	59.8	16592	92.70%
Termitomyces sp*(MG148)*	11375	86.5	18056	78.30%
Termitomyces sp*(MG16)*	9286	97.2	12480	81.40%
Thelephora aurantiotincta(MG58)	9868	28.6	13009	78.20%
Tricholoma bakamatsutake(MG51)	8907	142.3	14636	93.10%
Tricholoma flavovirens(MG32)	6710	122.3	21960	91.00%
Tricholoma matsutake(MG52)	8207	158	22912	92.40%
Tricholoma saponaceum (MG146)	11313	54.7	18973	86.90%
Tricholoma sp*(MG77)*	4575	120.5	26355	75.90%
Tricholoma terreum(MG45)	9244	83.9	23810	84.10%
Tricoloma sp*(razy-128)(MG99)*	8902	59.1	19530	85.20%
Tuber calosporum(MG102)	27301	166.8	13614	95.10%
Tuber microsphaerosporum(MG111)	32615	94.5	23533	94.40%
Tuber umbilicatum(MG104)	31265	81.4	17112	95.80%
Tylopilus plumbeoviolaceoides(MG33)	10852	48.8	21505	76.20%
Tylopilus virens(MG40)	10149	44.4	17464	75.50%
Xerocomus impolitus(MG39)	87675	40.4	11677	94.40%
