# Supplementary material for: The Genome Sequences of 90 Mushrooms
Source: Sci Rep. 2018 Jul 2;8:9982. doi: 10.1038/s41598-018-28303-2 (PMC6028375; doi:10.1038/s41598-018-28303-2)
Supplement: Supplementary file 5 — Table S3 [file 41598_2018_28303_MOESM5_ESM.pdf]

The Genome Sequences of 90 Mushrooms

Huiying Li[1], Surui Wu[3,#], Xiao Ma[2,4,5,#], Wei Chen[2,4], Jing Zhang[6], Shengchang Duan[6], Yun Gao[6], Ling Kui[7,8], Wenli Huang[12], Peng Wu[2,4], Ruoyu Shi[2,4], Yifan Li[2,5], Yuanzhong Wang[9], Jieqing Li[9], Xiang Guo[3], Xiaoli Luo[3], Qiang Li[12], Chuan Xiong[12], Honggao Liu[9], Mingying Gui[3*], Jun Sheng[2,4,*], Yang Dong[2,10,11,*]

[1]Kunming University of Science and Technology， Kunming, 650500, Yunnan, China.

[2]College of Biological Big Data, Yunnan Agriculture University,Kunming, 650201, Yunnan, China.

[3]Kunming Edible Fungi Institute of All China Federation of Supply and Marketing Cooperatives, Kunming , 650032, Yunnan, China

[4]Yunnan Research Institute for Local Plateau Agriculture and Industry, Kunming, 650201, Yunnan, China.

[5]Key Laboratory of Puer Tea Science, Ministry of Education, Yunnan Agricultural University, Kunming, 650201, Yunnan, China.

[6]Nowbio Biotechnology Company, Kunming, 650201, Yunnan, China.

[7]State Key Laboratory of Genetic Resources and Evolution, Kunming Institute of Zoology, Chinese Academy of Sciences, Kunming, 650223, Yunnan, China.

[8]Kunming College of Life Science, University of Chinese Academy of Sciences, Kunming 650204, Yunnan, China.

[9]College of Agronomy and Biotechnology, Yunnan Agricultural University, Kunming, 650201, Yunnan, China

[10]State Key Laboratory for Conservation and Utilization of Bio-Resources in Yunnan, Yunnan Agricultural University, Kunming, 650201, Yunnan, China.

[11]Key Laboratory for Agro-biodiversity and Pest Control of Ministry of Education, Yunnan Agricultural University, Kunming, 650201, Yunnan, China.

[12]Biotechnology and Nuclear Technology Research Institute, Sichuan Academy of Agricultural Sciences, Chengdu, 610061, Sichuan, China.

Supplementary Table S3:Summary of CAZymes and Microsatellites analysis.

#Huiying Li, Surui Wu, Xiao Ma contributed equally.

	CAZymes						SSR
	AA	CBM	CE	GH	GT	PL	number
Agrocybe cylindracea(MG21)	35	19	27	76	26	2	3439
Albatrellus ellisii(MG60)	17	8	12	25	14	1	13882
Albatrellus sp*(MG142)*	10	8	11	22	17	0	3820
Amanita pseudoporphyria(MG37)	12	10	9	18	26	2	2965
Annulohypoxylon stygium(MG137)	44	15	27	79	27	4	2293
Auricularia polytricha(MG66)	13	12	14	34	27	1	1374
Boletus bicolor(MG1)	13	17	19	54	42	1	2937
Boletus brunneissimus(MG7)	8	9	18	20	21	1	2349
Boletus calopus(MG23)	8	5	9	35	29	2	2171
Boletus edulis(MG6)	14	12	16	34	13	2	3692
Boletus magnificus(MG22)	8	8	8	29	17	0	2247
Boletus ornatipes(MG30)	8	4	8	30	21	0	2696
Boletus sp*(MG55)*	15	8	9	36	23	1	2398
Boletus sp*(razy-134)(MG95)*	16	10	14	29	14	1	2717
Boletus speciosus(MG10)	14	7	11	30	17	0	2687
Boletus subvelutipes(MG31)	14	7	6	26	23	0	3048
Butyriboletus roseoflavus(MG29)	11	7	6	25	16	1	2669
Cantharellus appalachiensis(MG38)	17	48	65	135	108	12	9679
Cantharellus cibarius(MG75)	14	21	48	92	60	7	3469
Cantharellus cinnabarinus(MG28)	12	3	6	35	14	1	5237
Chroogomphus rutilus(MG62)	22	10	10	36	23	2	20866

Collybia sp*(MG36)*	29	26	20	51	12	1	2932
Coprinus comatus(MG80)	33	10	20	61	25	3	5565
Craterellus lutescens(MG144)	36	145	141	324	161	29	8851
Gomphus bonarii(MG147)	14	10	14	36	18	0	10849
Gomphus sp*(MG54)*	24	13	20	42	15	1	21642
Grifola frondosa (MG88)	21	10	17	42	17	0	1424
Hygrophorus pudorinus (MG65)	10	10	12	29	19	2	7076
Hygrophorus russula(MG78)	7	6	12	32	15	1	1925
Hymenopellis chiangmaiae(MG56)	57	30	41	99	40	15	4037
Lactarius deliciosus(MG9)	9	9	12	34	16	1	4493
Lactarius echinatus (razy-131)(MG122)	17	11	10	23	20	2	4114
Lactarius hatsudake(MG20)	9	12	10	27	12	2	7242
Lactarius hygrophoroides(MG19)	14	9	9	24	16	0	5095
Lactarius indigo(rll-109)(MG109)	14	11	9	30	29	3	7218
Lactarius pinguis(MG27)	17	5	16	26	18	0	7383
Lactarius piperatus(MG49)	16	7	10	23	13	0	6815
Lactarius rugatus(rmsh-101)(MG108)	10	1	9	18	12	2	5167
Lactarius sp*(MG50)*	14	7	10	26	18	1	8950
Lactarius sp*(rll-107)(MG121)*	10	14	11	35	18	0	4825
Lactarius trivialis (MG71)	16	8	14	29	16	3	3193
Lactarius volemus(MG8)	15	5	13	26	19	2	6374
Laetiporus sulphureus(MG138)	12	13	19	52	14	2	1222
Macrolepiota dolichaula(MG24)	35	26	18	62	19	1	5354
Megacollybia marginata(MG68)	33	45	42	85	29	6	5195

Morchella eximia (MG90)	25	61	69	201	64	22	15141
Morchella septimelata(MG113)	20	9	14	50	16	3	18678
Morchella septimelata(MG91)	16	11	15	49	17	5	18422
Oudemansiella radicata(MG139)	45	24	37	93	31	3	3058
Pholiota microspora (MG134)	33	22	24	55	23	3	3098
Pleurotus citrinopileatus(MG63)	41	25	22	65	25	8	1845
Pleurotus eryngii var.tuoliensis(MG79)	29	25	17	62	19	7	1562
Pleurotus eryngii(MG61)	31	22	17	85	37	12	13071
Pleurotus platypus(MG11)	32	35	23	71	21	8	1588
Pulveroboletus ravenelii(MG41)	8	7	8	30	16	0	2212
Ramaria cf. rubripermanens(MG17)	17	6	15	47	22	0	5266
Ramaria sp*(MG151)*	19	8	11	39	24	3	9750
Russula abietina(MG43)	17	9	16	33	16	1	4911
Russula aff. compacta(MG44)	15	9	8	26	17	1	8838
Russula foetens(MG47)	14	3	10	35	25	3	5740
Russula lepida(MG46)	11	6	10	23	18	4	3195
Russula sp*(MG48)*	10	10	13	25	16	1	6554
Russula virescens(MG14)	19	6	10	26	17	0	4791
Sarcodon aspratus(MG57)	13	7	14	39	19	2	2833
Sarcodon sp*(razy-129)(MG97)*	12	9	12	41	14	0	3782
Schizophyllum commune(MG53)	30	32	36	109	27	11	1577
Stropharia rugosoannulata (MG69)	40	25	30	59	12	1	2900
Suillus alpinus(MG64)	18	5	14	38	18	2	1282
Suillus pictus(MG42)	15	10	17	48	25	2	3929

Suillus placidus(MG34)	12	10	9	31	16	3	1358
Suillus sp*(MG131)*	10	12	12	39	11	3	1405
Termitomyces eurrhizus(MG13)	13	12	14	52	20	5	6443
Termitomyces heimii(MG15)	13	15	20	54	17	5	4875
Termitomyces sp*(MG145)*	23	16	19	57	13	4	6451
Termitomyces sp*(MG148)*	19	11	17	44	12	5	5604
Termitomyces sp*(MG16)*	18	17	24	44	19	6	7732
Thelephora aurantiotincta(MG58)	6	2	5	24	13	2	1715
Tricholoma bakamatsutake(MG51)	19	3	8	22	18	2	11239
Tricholoma flavovirens(MG32)	13	6	17	24	14	3	13082
Tricholoma matsutake(MG52)	19	9	12	30	21	3	17900
Tricholoma saponaceum (MG146)	16	10	19	51	21	2	3320
Tricholoma sp*(MG77)*	12	8	17	36	18	2	10860
Tricholoma terreum(MG45)	19	7	10	36	22	3	6776
Tricoloma sp*(razy-128)(MG99)*	17	8	12	36	27	1	3632
Tuber calosporum(MG102)	13	9	13	22	19	1	30904
Tuber microsphaerosporum(MG111)	16	15	24	37	34	1	21493
Tuber umbilicatum(MG104)	15	10	9	24	17	1	17031
Tylopilus plumbeoviolaceoides(MG33)	18	8	12	26	28	0	2174
Tylopilus virens(MG40)	15	7	13	30	12	0	2005
Xerocomus impolitus(MG39)	14	10	12	35	11	1	2996
