# Supplementary material for: The Genome Sequences of 90 Mushrooms
Source: Sci Rep. 2018 Jul 2;8:9982. doi: 10.1038/s41598-018-28303-2 (PMC6028375; doi:10.1038/s41598-018-28303-2)
Supplement: Supplementary file 6 — Table S4 [file 41598_2018_28303_MOESM6_ESM.pdf]

The Genome Sequences of 90 Mushrooms

Huiying Li[1], Surui Wu[3,#], Xiao Ma[2,4,5,#], Wei Chen[2,4], Jing Zhang[6], Shengchang Duan[6], Yun Gao[6], Ling Kui[7,8], Wenli Huang[12], Peng Wu[2,4], Ruoyu Shi[2,4], Yifan Li[2,5], Yuanzhong Wang[9], Jieqing Li[9], Xiang Guo[3], Xiaoli Luo[3], Qiang Li[12], Chuan Xiong[12], Honggao Liu[9], Mingying Gui[3*], Jun Sheng[2,4,*], Yang Dong[2,10,11,*]

[1]Kunming University of Science and Technology, Kunming, 650500, Yunnan, China.

[2]College of Biological Big Data, Yunnan Agriculture University,Kunming, 650201, Yunnan, China.

[3]Kunming Edible Fungi Institute of All China Federation of Supply and Marketing Cooperatives, Kunming , 650032, Yunnan, China

[4]Yunnan Research Institute for Local Plateau Agriculture and Industry, Kunming, 650201, Yunnan, China.

[5]Key Laboratory of Puer Tea Science, Ministry of Education, Yunnan Agricultural University, Kunming, 650201, Yunnan, China.

[6]Nowbio Biotechnology Company, Kunming, 650201, Yunnan, China.

[7]State Key Laboratory of Genetic Resources and Evolution, Kunming Institute of Zoology, Chinese Academy of Sciences, Kunming, 650223, Yunnan, China.

[8]Kunming College of Life Science, University of Chinese Academy of Sciences, Kunming 650204, Yunnan, China.

[9]College of Agronomy and Biotechnology, Yunnan Agricultural University, Kunming, 650201, Yunnan, China

[10]State Key Laboratory for Conservation and Utilization of Bio-Resources in Yunnan, Yunnan Agricultural University, Kunming, 650201, Yunnan, China.

[11]Key Laboratory for Agro-biodiversity and Pest Control of Ministry of Education, Yunnan Agricultural University, Kunming, 650201, Yunnan, China.

[12]Biotechnology and Nuclear Technology Research Institute, Sichuan Academy of Agricultural Sciences, Chengdu, 610061, Sichuan, China.

#Huiying Li, Surui Wu, Xiao Ma contributed equally.

Supplementary Table S4:the accession of species in Mycobank.

species	Mycobank ID	species	Mycobank ID
Agrocybe cylindracea(MG21)	439297	*Megacollybia marginata(MG68)*	530323
Albatrellus ellisii(MG60)	325955	*Morchella eximia (MG90)*	355734
Amanita pseudoporphyria(MG37)	292458	*Morchella septimelata(MG113)*	563956
Auricularia polytricha(MG66)	156825	*Morchella septimelata(MG91)*	563956
Boletus bicolor (MG1)	206184	*Oudemansiella radicata(MG139)*	254292
Boletus brunneissimus(MG7)	284565	*Pholiota microspora (MG134)*	235533
Boletus calopus(MG23)	162779	*Pleurotus citrinopileatus(MG63)*	303973
Boletus edulis (MG6)	356530	*Pleurotus eryngii var.tuoliensis(MG79)*	133079
Boletus magnificus(MG22)	284588	*Pleurotus eryngii(MG61)*	170772
Boletus ornatipes(MG30)	273060	*Pleurotus platypus(MG11)*	166949
Boletus speciosus(MG10)	196130	*Pulveroboletus ravenelii(MG41)*	100667
Boletus subvelutipes(MG31)	170238	*Ramaria cf. rubripermanens(MG17)*	322282
Butyriboletus roseoflavus(MG29)	804788	*Russula abietina(MG43)*	168669
Cantharellus appalachiensis(MG38)	310365	*Russula aff. compacta(MG44)*	181887
Cantharellus cibarius(MG75)	200345	*Russula foetens(MG47)*	187177
Cantharellus cinnabarinus(MG28)	356863	*Russula lepida(MG46)*	209177
Chroogomphus rutilus(MG62)	328192	*Russula virescens(MG14)*	203449
Coprinus comatus(MG80)	148667	*Sarcodon aspratus(MG57)*	338801
Craterellus lutescens(MG144)	184644	*Stropharia rugosoannulata(MG69)*	145219
Gomphus bonarii(MG147)	286730	*Suillus alpinus(MG64)*	816992
Grifola frondosa (MG88)	362177	*Suillus pictus(MG42)*	586263

Hygrophorus pudorinus (MG65)	182455	*Suillus placidus(MG34)*	291280
Hygrophorus russula(MG78)	102423	*Termitomyces eurrhizus(MG13)*	549442
Hymenopellis chiangmaiae(MG56)	567949	*Termitomyces heimii(MG15)*	324479
Lactarius deliciosus(MG9)	224737	*Thelephora aurantiotincta(MG58)*	340026
Lactarius echinatus (razy-131)(MG122)	299296	*Tricholoma bakamatsutake(MG51)*	324912
Lactarius hatsudake(MG20)	223913	*Tricholoma flavovirens(MG32)*	340316
Lactarius hygrophoroides(MG19)	223547	*Tricholoma matsutake(MG52)*	307044
Lactarius indigo(rll-109)(MG109)	220303	*Tricholoma saponaceum (MG146)*	186494
Lactarius pinguis(MG27)	518945	*Tricholoma terreum(MG45)*	216171
Lactarius piperatus(MG49)	174964	*Tuber calosporum(MG102)*	814192
Lactarius rugatus(rmsh-101)(MG108)	299323	*Tuber microsphaerosporum(MG111)*	564356
Lactarius trivialis (MG71)	201199	*Tuber umbilicatum(MG104)*	357098
Lactarius volemus(MG8)	231293	*Tylopilus plumbeoviolaceoides(MG33)*	484972
Laetiporus sulphureus(MG138)	299348	*Tylopilus virens(MG40)*	340602
Macrolepiota dolichaula(MG24)	333540	*Xerocomus impolitus(MG39)*	499288
