# Supplementary material for: The Genome Sequences of 90 Mushrooms
Source: Sci Rep. 2018 Jul 2;8:9982. doi: 10.1038/s41598-018-28303-2 (PMC6028375; doi:10.1038/s41598-018-28303-2)
Supplement: Supplementary file 7 — Table S5 [file 41598_2018_28303_MOESM7_ESM.pdf]

The Genome Sequences of 90 Mushrooms

Huiying Li[1], Surui Wu[3,#], Xiao Ma[2,4,5,#], Wei Chen[2,4], Jing Zhang[6], Shengchang Duan[6], Yun Gao[6], Ling Kui[7,8], Wenli Huang[12], Peng Wu[2,4], Ruoyu Shi[2,4], Yifan Li [2,5], Yuanzhong Wang[9] , Jieqing Li[9] , Xiang Guo[3] , Xiaoli Luo[3], Qiang Li[12], Chuan Xiong[12], Honggao Liu[9], Mingying Gui[3*], Jun Sheng[2,4,*], Yang Dong[2,10,11,*]

[1]Kunming University of Science and Technology, Kunming, 650500, Yunnan, China.

[2]College of Biological Big Data, Yunnan Agriculture University,Kunming, 650201, Yunnan, China.

[3]Kunming Edible Fungi Institute of All China Federation of Supply and Marketing Cooperatives, Kunming , 650032, Yunnan, China

[4]Yunnan Research Institute for Local Plateau Agriculture and Industry, Kunming, 650201, Yunnan, China.

[5]Key Laboratory of Puer Tea Science, Ministry of Education, Yunnan Agricultural University, Kunming, 650201, Yunnan, China.

[6]Nowbio Biotechnology Company, Kunming, 650201, Yunnan, China.

[7]State Key Laboratory of Genetic Resources and Evolution, Kunming Institute of Zoology, Chinese Academy of Sciences, Kunming, 650223, Yunnan, China.

[8]Kunming College of Life Science, University of Chinese Academy of Sciences, Kunming 650204, Yunnan, China.

[9]College of Agronomy and Biotechnology, Yunnan Agricultural University, Kunming, 650201, Yunnan, China

[10]State Key Laboratory for Conservation and Utilization of Bio-Resources in Yunnan, Yunnan Agricultural University, Kunming, 650201, Yunnan, China.

[11]Key Laboratory for Agro-biodiversity and Pest Control of Ministry of Education, Yunnan Agricultural University, Kunming, 650201, Yunnan, China.

[12]Biotechnology and Nuclear Technology Research Institute, Sichuan Academy of Agricultural Sciences, Chengdu, 610061, Sichuan, China.

#Huiying Li, Surui Wu, Xiao Ma contributed equally.

Supplementary Table S5:Statistics of the coverage of the genome.

species	Coverage(X)	species	Coverage(X)
Agrocybe chaxinggu(MG21)	76	*Morchella eximia (MG90)*	57
Albatrellus ellisii(MG60)	67	*Morchella septimelata(MG113)*	88
Albatrellus sp*(MG142)*	32	*Morchella septimelata(MG91)*	58
Amanita pseudoporphyria(MG37)	90	*Oudemansiella radicata(MG139)*	26
Annulohypoxylon stygiun(MG137)	117	*Pholiota microspora (MG134)*	49
Auricularia polytricha(MG66)	147	*Pleurotus citrinopileatus(MG63)*	95
Boletus bicolor(MG1)	65	*Pleurotus eryngii var.tuoliensis(MG79)*	77
Boletus brunneissimus(MG7)	95	*Pleurotus eryngii(MG61)*	58
Boletus calopus(MG23)	102	*Pleurotus platypus(MG11)*	60
Boletus edulis(MG6)	67	*Pulverboletus ravenelii(MG41)*	78
Boletus magnificus(MG22)	94	*Ramaria cf. rubripermanens(MG17)*	74
Boletus ornatipes(MG30)	79	*Ramaria* sp*(MG151)*	96
Boletus sp *(razy-134)(MG95)*	90	*Russula abietina(MG43)*	81
Boletus sp *(MG55)*	93	*Russula aff. compacta(MG44)*	58
Boletus speciosus(MG10)	88	*Russula foetens(MG47)*	78
Boletus subvelutipes(MG31)	116	*Russula lepida(MG46)*	85
Butyriboletus roseoflavus(MG29)	96	*Russula* sp*(MG48)*	46
Cantharellus appalachiensis(MG38)	91	*Russula virescens(MG14)*	48
Cantharellus cibarius(MG75)	112	*Sarcodon aspratum(MG57)*	79
Cantharellus cinnabarinus(MG28)	93	*Sarcodon* sp *(razy-129)(MG97)*	116
Chroogomphus rutilus(MG62)	73	*Schizophyllum commune(MG53)*	122

Collybia sp*(MG36)*	71	*Stropharia rugosoannulata (MG69)*	51
Coprinus comatus(MG80)	122	*Suillus alpinus(MG64)*	109
Craterellus lutescens(MG144)	94	*Suillus pictus(MG42)*	81
Gomphus bonarii(MG147)	72	*Suillus placidus(MG34)*	91
Gomphus sp*(MG54)*	59	*Suillus* sp*(MG131)*	71
Grifola frondosa (MG88)	132	*Termitomyces eurrhizus(MG13)*	101
Hygrophorus pudorinus (MG65)	68	*Termitomyces heimii(MG15)*	65
Hygrophorus russula(MG78)	114	*Termitomyces* sp*(MG148)*	64
Hymenopellis chiangmaiae(MG56)	74	*Termitomyces* sp*(MG16)*	119
Lactarius deliciosus(MG9)	82	*Termitomyces* sp.*(MG145)*	102
Lactarius echinetus (razy-131)(MG122)	108	*Thelephora aurantiotincta(MG58)*	160
Lactarius hatsudake(MG20)	85	*Tricholoma bakamatsutake(MG51)*	68
Lactarius hygrophoroides(MG19)	83	*Tricholoma flavovirens(MG32)*	66
Lactarius indigo(rll-109)(MG109)	46	*Tricholoma matsutake(MG52)*	73
Lactarius orange（rll-107)(MG121)	112	*Tricholoma saponaceum (MG146)*	87
Lactarius pinguis(MG27)	104	*Tricholoma* sp *(MG77)*	41
Lactarius piperatus(MG49)	72	*Tricholoma terreum(MG45)*	75
Lactarius rugatus(rmsh-101)(MG108)	119	*Tricoloma* sp *(razy-128)(MG99)*	71
Lactarius sp*(MG50)*	72	*Tuber calosporum(MG102)*	84
Lactarius trivialis (MG71)	104	*Tuber microsphaerosporum(MG111)*	151
Lactarius volemus(MG8)	87	*Tuber umbilicatum(MG104)*	119
Laetiporus sulphureus(MG138)	90	*Tylopilus plumbeoviolaceoides(MG33)*	100
Macrolepiota dolichaula(MG24)	146	*Tylopilus virens(MG40)*	82
Megacollybia marginata(MG68)	88	*Xerocomus impolitus(MG39)*	81
