# Supplementary material for: The Genome Sequences of 90 Mushrooms
Source: Sci Rep. 2018 Jul 2;8:9982. doi: 10.1038/s41598-018-28303-2 (PMC6028375; doi:10.1038/s41598-018-28303-2)
Supplement: Supplementary file 8 — Table S6 [file 41598_2018_28303_MOESM8_ESM.pdf]

The Genome Sequences of 90 Mushrooms

Huiying Li[1], Surui Wu[3,#]，Xiao Ma[2,4,5,#], Wei Chen[2,4], Jing Zhang[6], Shengchang Duan[6], Yun Gao[6], Ling Kui[7,8], Wenli Huang[12], Peng Wu[2,4]，Ruoyu Shi[2,4]，Yifan Li [2,5], Yuanzhong Wang[9] , Jieqing Li[9] , Xiang Guo[3] , Xiaoli Luo[3], Qiang Li[12], Chuan Xiong[12], Honggao Liu[9], Mingying Gui[3*], Jun Sheng[2,4,*], Yang Dong[2,10,11,*]

[1]Kunming University of Science and Technology，Kunming, 650500, Yunnan, China.

[2]College of Biological Big Data, Yunnan Agriculture University,Kunming, 650201, Yunnan, China.

[3]Kunming Edible Fungi Institute of All China Federation of Supply and Marketing Cooperatives, Kunming , 650032, Yunnan, China

[4]Yunnan Research Institute for Local Plateau Agriculture and Industry, Kunming, 650201, Yunnan, China.

[5]Key Laboratory of Puer Tea Science, Ministry of Education, Yunnan Agricultural University, Kunming, 650201, Yunnan, China.

[6]Nowbio Biotechnology Company, Kunming, 650201, Yunnan, China.

[7]State Key Laboratory of Genetic Resources and Evolution, Kunming Institute of Zoology, Chinese Academy of Sciences, Kunming, 650223, Yunnan, China.

[8]Kunming College of Life Science, University of Chinese Academy of Sciences, Kunming 650204, Yunnan, China.

[9]College of Agronomy and Biotechnology, Yunnan Agricultural University, Kunming, 650201, Yunnan, China

[10]State Key Laboratory for Conservation and Utilization of Bio-Resources in Yunnan, Yunnan Agricultural University, Kunming, 650201, Yunnan, China.

[11]Key Laboratory for Agro-biodiversity and Pest Control of Ministry of Education, Yunnan Agricultural University, Kunming, 650201, Yunnan, China.

[12]Biotechnology and Nuclear Technology Research Institute, Sichuan Academy of Agricultural Sciences, Chengdu, 610061, Sichuan, China.

#Huiying Li, Surui Wu, Xiao Ma contributed equally.

Supplementary Table S6:The Project ID of raw data in NCBI.

**Species**	project ID	**Species**	project ID
Agrocybe cylindracea(MG21)	PRJNA392296	*Morchella eximia (MG90)*	PRJNA392557
Albatrellus ellisii(MG60)	PRJNA392535	*Morchella septimelata(MG113)*	PRJNA392573
Albatrellus sp*(MG142)*	PRJNA392575	*Morchella septimelata(MG91)*	PRJNA392555
Amanita pseudoporphyria(MG37)	PRJNA392512	*Oudemansiella radicata(MG139)*	PRJNA392576
Annulohypoxylon stygiun(MG137)	PRJNA392572	*Pholiota microspora (MG134)*	PRJNA392569
Auricularia polytricha(MG66)	PRJNA392540	*Pleurotus citrinopileatus(MG63)*	PRJNA392544
Boletus bicolor(MG1)	PRJNA391764	*Pleurotus eryngii var.tuoliensis(MG79)*	PRJNA392551
Boletus brunneissimus(MG7)	PRJNA392235	*Pleurotus eryngii(MG61)*	PRJNA392536
Boletus calopus(MG23)	PRJNA392499	*Pleurotus platypus(MG11)*	PRJNA392246
Boletus edulis(MG6)	PRJNA392229	*Pulverboletus ravenelii(MG41)*	PRJNA392513
Boletus magnificus(MG22)	PRJNA392497	*Ramaria cf. rubripermanens(MG17)*	PRJNA392293
Boletus ornatipes(MG30)	PRJNA392504	*Ramaria* sp*(MG151)*	PRJNA391778
Boletus sp *(razy-134)(MG95)*	PRJNA392554	*Russula abietina(MG43)*	PRJNA392517
boletus sp *(MG55)*	PRJNA392530	*Russula aff. compacta(MG44)*	PRJNA392520
Boletus speciosus(MG10)	PRJNA392242	*Russula foetens(MG47)*	PRJNA392519
Boletus subvelutipes(MG31)	PRJNA392502	*Russula lepida(MG46)*	PRJNA392521
Butyriboletus roseoflavus(MG29)	PRJNA392505	*Russula* sp*(MG48)*	PRJNA392523
Cantharellus appalachiensis(MG38)	PRJNA392508	*Russula virescens(MG14)*	PRJNA392250
Cantharellus cibarius(MG75)	PRJNA392548	*Sarcodon aspratum(MG57)*	PRJNA392533
Cantharellus cinnabarinus(MG28)	PRJNA392498	*Sarcodon* sp *(razy-129)(MG97)*	PRJNA392563
Chroogomphus rutilus(MG62)	PRJNA392538	*Schizophyllum commune(MG53)*	PRJNA392531

Collybia sp*(MG36)*	PRJNA392511	*Stropharia rugosoannulata (MG69)*	PRJNA392541
Coprinus comatus(MG80)	PRJNA392549	*Suillus alpinus(MG64)*	PRJNA392543
Craterellus lutescens(MG144)	PRJNA392577	*Suillus pictus(MG42)*	PRJNA392514
Gomphus bonarii(MG147)	PRJNA392578	*Suillus placidus(MG34)*	PRJNA392507
Gomphus sp*(MG54)*	PRJNA392529	*Suillus* sp*(MG131)*	PRJNA392568
Grifola frondosa (MG88)	PRJNA392553	*Termitomyces eurrhizus(MG13)*	PRJNA392247
Hygrophorus pudorinus (MG65)	PRJNA392537	*Termitomyces heimii(MG15)*	PRJNA392254
Hygrophorus russula(MG78)	PRJNA392546	*Termitomyces* sp*(MG148)*	PRJNA392580
Hymenopellis chiangmaiae(MG56)	PRJNA392532	*Termitomyces* sp*(MG16)*	PRJNA392288
Lactarius deliciosus(MG9)	PRJNA392238	*Termitomyces* sp*(MG145)*	PRJNA392581
Lactarius echinetus (razy-131)(MG122)	PRJNA392571	*Thelephora aurantiotincta(MG58)*	PRJNA392534
Lactarius hatsudake(MG20)	PRJNA392289	*Tricholoma bakamatsutake(MG51)*	PRJNA392524
Lactarius hygrophoroides(MG19)	PRJNA392295	*Tricholoma flavovirens(MG32)*	PRJNA392503
Lactarius indigo(rll-109)(MG109)	PRJNA392565	*Tricholoma matsutake(MG52)*	PRJNA392528
Lactarius orange（rll-107)(MG121)	PRJNA392566	*Tricholoma saponaceum (MG146)*	PRJNA392579
Lactarius pinguis(MG27)	PRJNA392496	*Tricholoma* sp *(MG77)*	PRJNA392550
Lactarius piperatus(MG49)	PRJNA392527	*Tricholoma terreum(MG45)*	PRJNA392518
Lactarius rugatus(rmsh-101)(MG108)	PRJNA392564	*Tricoloma* sp *(razy-128)(MG99)*	PRJNA392562
Lactarius sp*(MG50)*	PRJNA392525	*Tuber calosporum(MG102)*	PRJNA392559
Lactarius trivialis (MG71)	PRJNA392547	*Tuber microsphaerosporum(MG111)*	PRJNA392561
Lactarius volemus(MG8)	PRJNA392221	*Tuber umbilicatum(MG104)*	PRJNA392560
Laetiporus sulphureus(MG138)	PRJNA392570	*Tylopilus plumbeoviolaceoides(MG33)*	PRJNA392506
Macrolepiota dolichaula(MG24)	PRJNA392501	*Tylopilus virens(MG40)*	PRJNA392515
Megacollybia marginata(MG68)	PRJNA392542	*Xerocomus impolitus(MG39)*	PRJNA392510
